# Direct cortical stimulation–evoked symptoms in the human temporal pole: a cohort study

**DOI:** 10.1016/j.cnp.2026.05.004

**Published:** 2026-05-21

**Authors:** Yulia Novitskaya, Andreas Schulze-Bonhage

**Affiliations:** Epilepsy Center, Department of Neurosurgery, Faculty of Medicine, University of Freiburg, Breisacher Str. 64, Freiburg 79106, Germany

**Keywords:** Temporal pole, Direct cortical stimulation, Anatomical-functional relationships

## Abstract

**Objective:**

The temporal pole is a part of temporolimbic networks and may contribute to seizure generation in temporal lobe epilepsy. Direct cortical stimulation (DCS) during invasive presurgical evaluation in epilepsy patients provides information about its functional organization.

**Methods:**

We retrospectively analyzed DCS data from epilepsy patients at a tertiary center between 2012 and 2025. Patients with at least one temporopolar DCS session were included. Stimulation-induced symptoms were categorized by modality and frequency.

**Results:**

Fifty patients (58% male, median age 33 years) underwent temporopolar DCS, comprising 157 stimulation trials. Overall, 66% of patients reported at least one symptom across a broad range of modalities. Sensory and motor manifestations were most frequent (75%). Although much less common (24%), cognitive, emotional, and visceral symptoms were significantly more likely to resemble patients' habitual seizure semiology (GLMM, OR = 13.0, 95% CI 2.42–69.7, *p* = 0.003). Thirteen patients reported ipsilateral painful facial sensations, likely reflecting activation of trigeminal sensory pathways due to current spread near the skull base.

**Conclusions:**

Temporopolar DCS elicits heterogeneous symptoms, supporting its role as an integrative network node rather than a region with distinct stimulation-induced semiology.

**Significance:**

The findings limit the localizing value of temporopolar DCS responses in presurgical evaluation.

## Highlights

• High symptom variability with frequent sensory effects.
• Cognitive, emotional and visceral symptoms are rare but resemble habitual seizures.
• Painful facial sensations due to activation of ipsilateral trigeminal pathways.

## 1 Introduction

The temporal pole (Brodmann area 38) is a cortical structure found exclusively in non-human primates, great apes, and humans. It constitutes the rostral portion of the temporal lobe and lacks a distinct sulcus or other reliable landmarks on the cortical surface, making its anatomical boundaries difficult to delineate (Chabardès et al., 2002). The temporal pole is densely interconnected with mesial temporal structures, the limbic system, visual and auditory association cortices, as well as orbitofrontal and medial frontal regions (Morán et al., 1987; Mohedano-Moriano et al., 2007; Ng et al., 2014; Muñoz-López et al., 2015; Novitskaya et al., 2020; Mesulam, 2023). Functionally, the anterior temporal cortex, including the temporal pole, has been implicated in a broad range of higher-order cognitive processes, such as semantic processing (Schwen Blackett et al., 2022; Tiesinga et al., 2023), face (Deen et al., 2024) and voice recognition (Andics et al., 2010; Ohki et al., 2016), social cognition (Olson et al., 2007), and naming (Tranel, 2009; Abel et al., 2015).

Temporal lobe epilepsy (TLE) is the most common form of focal epilepsy referred for surgical treatment and is frequently refractory to antiseizure medications (Téllez-Zenteno and Hernández-Ronquillo, 2012). However, the prevalence of epilepsy with seizure onset specifically in the temporal pole remains poorly characterized, as most epidemiological studies of TLE do not delineate the precise site of seizure initiation within the temporal lobe. Early stereoelectroencephalography (SEEG) studies have reported temporal pole involvement in approximately 50% of patients, either occurring simultaneously with or preceding hippocampal seizure activity (Kahane et al., 2002; Chabardès et al., 2005).

Direct cortical stimulation (DCS) of the human brain, delivered using low-frequency (1 Hz) or high-frequency (50 Hz) electrical currents, is a standard technique employed during invasive presurgical evaluation in patients with drug-resistant epilepsy and has been in clinical use for several decades (Trébuchon and Chauvel, 2016). DCS is used to help delineate the seizure onset zone (SOZ) as well as the symptomatogenic zone by reproducing clinical signs and/or electrographic features characteristic of a patient's habitual seizures (Schulz et al., 1997). Although DCS demonstrates high specificity (96.9%) for SOZ identification, its sensitivity remains low (23.0%), largely due to a substantial rate of false-negative stimulations within epileptogenic regions (Ley et al., 2022).

Most large-scale SEEG/DCS studies have focused on mesial temporal structures, such as the hippocampus, amygdala, and parahippocampal gyrus, rather than the temporal pole. Consequently, data specifically addressing temporopolar stimulation are limited and often underreported. In the present study, we aim to address this gap by providing additional clinical observations focused on the temporal pole.

## 2 Methods

This study analyzed intracranial SEEG data collected between January 2012 and December 2025 during invasive presurgical evaluations at the tertiary Epilepsy Center of the Freiburg University Hospital, Germany. A total of 219 pediatric and adult patients with epilepsy who underwent intracranial SEEG recordings using depth electrodes were identified. Among these patients, 53% (117/219) had a SEEG depth electrode implanted in the temporal pole, and DCS of the temporal pole was performed in 50 patients, corresponding to 23% (50/219) of the total cohort.

Indications for intracranial SEEG, selection of electrode implantation sites, and DCS procedures were determined exclusively by clinical considerations aimed at localizing and delineating the seizure onset zone. Intracerebral electrodes contained 4–12 contiguous contacts consisting of cylinders of an area of 5.03 mm^2^ (Microdeep depth electrode, Dixi Medical, France), or 6.53 mm^2^ (Epilepsy/LTM Behnke Fried depth electrode, Ad-Tech Medical Instrument Corporation, USA), or 8.29 mm^2^ (Epilepsy/LTM Spencer Probe depth electrodes Ad-Tech Medical Instrument Corporation, USA).

**Fig. 1:**
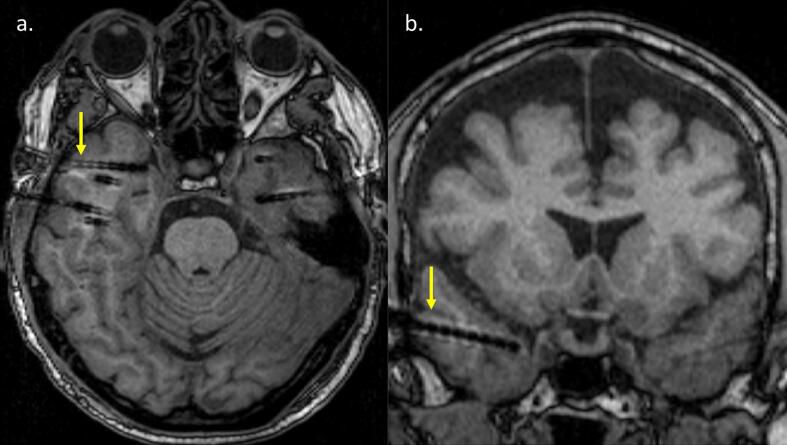
Axial (a) and coronal (b) T1-weighted MPRAGE images showing typical placement of a depth electrode in the right temporal pole (arrows) in a representative patient.

After the stereotactic placement of electrodes had been completed, each patient underwent a post-operative 3D T1-MPRAGE MRI scan for visual determining precise anatomical gyral and sulcal position of each contact (an example of a SEEG depth electrode positioned in the right temporal pole is given in Fig. 1). The temporopolar region was delineated as the rostral portion of the inferior temporal, occipitotemporal, and superior temporal sulci on the lateral and inferior surfaces. On the medial surface, its posterior limit corresponded to the rhinal sulcus, an anterior and medial extension of the collateral sulcus (Chabardès et al., 2002).

**Fig. 2:**
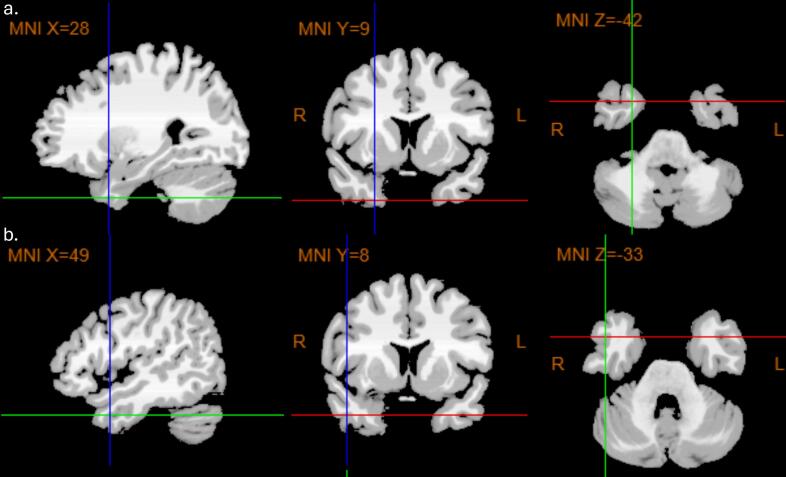
Localization of mesial (a) and lateral (b) depth electrode contacts based on their median MNI coordinates, identified across all participants. The positions of both mesial and lateral contacts correspond to Brodmann area 38.

Depth electrodes targeting the temporal pole typically comprised up to 8–9 recording contacts. The four most distal contacts within the temporal pole were classified as mesial temporal pole, whereas the more proximal contacts were assigned to the lateral temporal pole. For the most distal (mesial) contacts, the following median MNI coordinates with interquartile range (IQR) were detected across the participants: X = 28 (IQR 5), Y = 9 (IQR 7.5), Z = −42 (IQR 7.5), corresponding to Brodmann area 38. The proximal contacts, ranging from the 5th to the 9th contact from the electrode tip, yielded the following median MNI coordinates: X = 49 (IQR 7), Y = 7.5 (IQR 8.3), Z = −33 (10.3), also corresponding to Brodmann area 38 (Fig. 2).

DCS was administered via two adjacent contacts in a bipolar configuration using biphasic square-wave pulses, delivered either at low-frequency (1 Hz, pulse width 2000 μs) or high-frequency (50 Hz, pulse width 1000 μs), in accordance with established clinical protocols (Trébuchon and Chauvel, 2016). The number of stimulated sites within the temporal pole varied across patients based on clinical considerations. Typically, each site within the temporal pole was stimulated once for a given set of stimulation parameters; repeated DCS trials at the same site were counted as a single stimulation.

Stimulation intensity was gradually up-titrated to a maximum of 15 mA in 1–3 mA increments for low-frequency stimulation and to a maximum of 6 mA in 0.5 mA increments for high-frequency stimulation. In cases of spread or persistent afterdischarges, stimulation of the electrode contacts was terminated to avoid DCS-induced seizures.

Both low-frequency (1 Hz) and high-frequency (50 Hz) stimulation were performed as part of routine clinical care. Consequently, retrospective analysis of these data did not require additional informed consent or ethical board approval.

Study outcomes comprised demographic and clinical characteristics, as well as patient-reported symptoms elicited during DCS. Symptoms were extracted from synchronized video recordings by two board-certified epileptologists (YN, ASB). Outcomes were analyzed using descriptive statistics. Proportions are presented with 95% exact binomial confidence intervals (CI), while continuous and count variables are summarized as median and interquartile range (IQR). The association between symptom type, habitual recognition, site of stimulation and the occurrence of afterdischarges was assessed using a generalized linear mixed-effects model (GLMM) with a binomial distribution and logit function. A patient-specific random intercept was included to account for clustering of stimulation trials within individuals.

## 3 Results

### 3.1 Demographic data and stimulation parameters

The DCS study cohort comprised 50 patients, including 29 males (58%). Patient age ranged from 6 to 57 years (median 33 years, IQR 23), with adults (≥18 years) accounting for 86% (95% CI: 76–96%) of the cohort. A temporal lobe seizure origin was confirmed in 39 patients (78%, 95% CI: 66–90%), whereas an extratemporal seizure origin was identified in 11 patients (22%, 95% CI: 10–34%). In 17 patients (34%, 95% CI: 20–48%), the seizure onset zone was partially or entirely localized to the temporal pole.

A total of 157 stimulations were delivered to the temporal pole, with 61% applied to the right hemisphere. 50 Hz-stimulation was performed in 112 temporopolar sites (71%, 95% CI: 64–78%), whereas 1 Hz-stimulation was applied in 45 sites (29%, 95% CI: 22–36%). The applied current intensity ranged from 1 to 6 mA (median 3 mA, IQR 1.5) for 50 Hz-DCS and from 1 to 15 mA (median 10 mA, IQR 5) for 1 Hz-DCS. The simulation duration varied from 1.5 to 10 s (median 3 s, IQR 2) for 50-Hz DCS and from 2 to 45 s (median 8 s, IQR 8.5) for 1 Hz-DCS.

Clinical symptoms of any modality were observed in 63 DCS trials (40%, 95% CI: 32–48%). Of these, 47 trials involved stimulation of the mesial temporal pole (74%, 95% CI: 64–86%), whereas the more lateral part of the temporal pole was stimulated in 16 trials (25%, 95% CI: 14–36%).

Afterdischarges, transient SEEG changes following electrical brain stimulation, characterized by a burst of epileptiform activity (Curot et al., 2017), occurred in 57 DCS trials (36%, 95% CI: 29–44%) and were mainly local to regional. In one case, temporopolar stimulation induced a clinical seizure, preceded by a SEEG seizure pattern originating in the ipsilateral hippocampus.

### 3.2 Symptoms semiology

Thirty-three of 50 patients (66%, 95% CI: 52–79%) experienced at least one stimulation-induced symptom. The number of stimulation-induced symptoms per patient ranged from 1 to 5 (median 1, IQR 2). 17 patients recognized the elicited symptoms as fully or partially habitual (34%, 95% CI: 20–47%).

Habitual symptoms elicited exclusively during temporopolar DCS were observed in only 2 of 17 patients (12%). In both cases, these were described as undefinable sensations. Among the remaining patients, qualitatively similar symptoms, typically differing in intensity and/or occurring in combination with additional symptoms, were elicited by stimulation of adjacent structures, most commonly the entorhinal cortex and amygdala, in 10 patients (59%). In 4 patients (24%), comparable symptoms were induced by stimulation of the ipsilateral hippocampus and parahippocampal gyrus. In one patient, similar symptoms were observed during stimulation of the ipsilateral insula.

Reported symptoms were classified into five categories based on modality: sensory, cognitive, emotional or psychic, visceral or autonomic, and motor. An overview of symptoms and occurrence rates is provided in Table 1.

**Table 1:** Clinical symptoms elicited by direct cortical stimulation (DCS) of the human temporal pole.

Symptoms category	Symptoms description	Reported symptoms, n	Part of typical seizures, n	AD overall/ in habitual symptoms, n	SOZ, within/ outside, n	Side of DCS, R/ L, n	Site of DCS, mes./ lat., n	DCS current 50 Hz/ 1 Hz, n
**Sensory**								
visual	shaky image, flash of light, tilted image	4	0	0 / 0	2 / 2	4 / 0	4 / 0	1 / 3
skin sensation/ somatosensory	hypaesthesia or paraesthesia of head, face, hands or legs	9	3	3 / 2	3 / 6	7 / 2	4 / 5	5 / 4
vestibular	feeling of tilting to the side, feeling of pulling down, vertigo	4	2	2 / 2	1 / 3	3 / 1	3 / 1	3 / 1
auditory	ringing in the ear	1	0	0 / 0	0 / 1	1 / 0	0 / 1	0 / 1
painful sensation	electric shock-like feeling in the head, pain in the face, palate or teeth, throbbing feeling, headache	13	0	3 / 0	4 / 9	7 / 6	8 / 5	5 / 8
undefinable sensation	“strange” feeling, indescribable feeling	3	3	2 / 2	1 / 2	2 / 1	3 / 0	3 / 0
**Cognitive**	memory flashbacks, déjà vu	4	3	3 / 2	1 / 3	1 / 3	4 / 0	4 / 0
	difficulty thinking, “brain fog”	3	2	0 / 0	2 / 1	1 / 2	3 / 0	2 / 1
**Emotional/psychic**	inner tension	1	1	1 / 1	1 / 0	1 / 0	1 / 0	1 / 0
	fear	1	1	1 / 1	0 / 1	1 / 0	1 / 0	1 / 0
	sense of depersonalization	1	1	0 / 0	0 / 1	0 / 1	1 / 0	1 / 0
**Visceral/autonomic**	ascending epigastric sensation	4	3	4 / 3	1 / 3	3 / 1	4 / 0	4 / 0
	constriction or tension in the chest	4	4	2 / 2	3 / 1	3 / 1	4 / 0	4 / 0
	heat sensation	1	1	1 / 1	1 / 0	1 / 0	1 / 0	1 / 0
	sweating	1	0	1 / 0	0 / 1	0 / 1	0 / 1	1 / 0
	nausea	1	1	1 / 1	0 / 1	1 / 0	1 / 0	1 / 0
	tachycardia	1	1	1 / 1	1 / 0	1 / 0	1 / 0	0 / 1
**Motor**	facial twitching	5	0	0 / 0	2 / 3	4 / 1	4 / 1	1 / 4
	polytopic myoclonic twitching	2	1	0 / 0	0 / 2	1 / 1	2 / 0	1 / 1

Values represent the number of patients (n), counted once per patient regardless of the number of stimulation trials.

AD: afterdischarges, DCS: direct current stimulation, L: left, lat.: lateral, mes.: mesial, R: right, SOZ: seizure onset zone.

Consistent with the established semiology of temporal lobe seizures, 12 patients (24%) in our cohort reported cognitive, emotional, and visceral or autonomic symptoms in 15 DCS trials (24%, 95% CI: 13–35%). Sensory symptoms of different submodalities were observed more frequently, in 47 DCS trials (75%, 95% CI: 64–86%). Among sensory symptoms, visual, auditory and vestibular signs as well as transient somatosensory sensation including painful sensation were reported. Motor symptoms included facial twitching and polytopic myoclonic twitching were induced in 6 DCS trials (10%, 95% CI: 2–17%). No behavioral symptoms were observed in this cohort.

Despite being infrequently elicited, temporal symptoms (i.e., cognitive, emotional, and visceral/autonomic manifestations) were significantly more likely to be identified as habitual compared with sensory and motor symptoms (GLMM, OR = 13.0, 95% CI 2.42–69.7, *p* = 0.003). A trend toward an association was observed between the occurrence of afterdischarges on SEEG and symptoms identified by patients as habitual; however, this did not reach statistical significance (OR = 3.6, 95% CI 0.88–14.8, *p* = 0.074).

Furthermore, no association was found between DCS trials targeting the more mesial portion of the temporal pole and the occurrence of temporal semiology (OR = 2.5, 95% CI 0.39–16.4, *p* = 0.322). Similarly, sensory and motor symptoms were not elicited by stimulation of either the more mesial temporal pole (OR = 0.7, 95% CI 0.09–4.9, *p* = 0.694) or the more lateral temporal pole (OR = 1.5, 95% CI 0.2–10.7, p = 0.694).

### 3.3 Painful sensation

Thirteen patients (26%, 13/50) reported painful sensations during 17 DCS trials (27%, 95% CI: 16–38%). Pain was predominantly localized to the head and face and was not recognized as part of the patients' habitual seizure semiology (OR = 0.04, 95% CI: 0.003–0.58, *p* = 0.019). Eight patients (16%, 8/50) described facial pain characterized by brief, electric shock–like sensations localized to the cheek and the area around the corner of the mouth, including dental pain in three cases. The pain was consistently ipsilateral to the side of stimulation and persisted for the entire duration of DCS. Similarly, five patients (10%, 5/50) reported DCS-induced facial twitching confined to the ipsilateral corner of the mouth. Notably, painful sensations were not associated with stimulation of the more mesial portion of the temporal pole (OR = 1.9, 95% CI: 0.4–8.1, *p* = 0.381).

Stimulation parameters associated with painful sensations were within standard limits. For low-frequency (1 Hz) stimulation, current intensity ranged from 1 to 10 mA (median 5 mA, IQR 5.8), with a stimulation duration of 2–15 s (median 5 s, IQR 2.5). For high-frequency (50 Hz) stimulation, current intensity ranged from 1 to 6 mA (median 2.5 mA, IQR 1.5), with a stimulation duration of 2–5 s (median 3 s, IQR 0.5).

## 4 Discussion

Temporal lobe epilepsy, the most common form of epilepsy, is associated with a broad range of clinical manifestations, including sensory, cognitive, emotional, autonomic, and behavioral symptoms (Frazzini et al., 2022; Schulze-Bonhage and San Antonio-Arce, 2025). Many of these responses have been replicated in DCS studies of the mesial temporal lobe performed during invasive presurgical evaluation (Wieser, 1983; Fish et al., 1993; Inman et al., 2020; Soulier et al., 2023; Hao et al., 2023). Studies involving DCS of the temporal pole suggest that stimulation of this region evokes a comparably rich symptomatology.

Just as the temporal pole lacks a clear anatomical demarcation, no specific seizure semiology can be attributed exclusively to this region. Both the existing literature and our own DCS data indicate that stimulation of the temporal pole is associated with a wide range of clinical manifestations. Moreover, these DCS-elicited symptoms substantially overlap with those induced by stimulation of adjacent and interconnected regions, including amygdala, hippocampus, uncus, parahippocampal gyrus, and the lateral temporal cortex (Mulak et al., 2008; Mariani et al., 2021).

Visceral sensations and psychic phenomena represent some of the most common components of temporal lobe seizure semiology. Accordingly, affective responses to temporopolar stimulation have been previously observed and described as predominantly dysphoric, manifesting as fear, anxiety, or sadness (Ostrowsky et al., 2002; Meletti et al., 2006; Soulier et al., 2023), consistent with our own observations. In a large-scale study involving 203 patients and 115 mesial temporal stimulations, including the temporal pole, no positive emotional responses were elicited (Soulier et al., 2023). This predominance of negative affect aligns with clinical observations that ictal fear is a common feature of mesial temporal lobe epilepsy (Bancaud et al., 1994; Perven and So, 2015; Khoja et al., 2021). In contrast, positive emotional responses during temporopolar DCS have been reported only rarely, including sensations of well-being or relaxation in a single patient (Ostrowsky et al., 2002), happiness or joy in four patients (Meletti et al., 2006), and excitement accompanied by an urge to laugh in a few cases (Gordon et al., 1996).

Visceral sensations, the conscious perception of internal bodily signals, are common in temporal lobe epilepsy and most frequently manifest as abdominal or “epigastric” auras, reported in up to 73% of patients (Henkel et al., 2002; Perven and So, 2015). These phenomena also encompass viscero-sensitive sensations (e.g., perceptions arising from the throat, thorax, or abdomen) as well as viscero-vegetative symptoms, including flushing, nausea, tachycardia, and dyspnea (Soulier et al., 2023). Although visceral sensations have been reported during DCS of the temporal pole in a limited number of studies, this region appears to be less strongly associated with their induction than the amygdala or hippocampus (Mariani et al., 2021; Soulier et al., 2023). In our cohort, viscero-sensitive sensations and viscero-vegetative (autonomic) symptoms were commonly reported as components of patients' habitual seizures and were frequently associated with afterdischarges.

Among other semiologies, DCS of the temporal pole have been observed to elicit sensory symptoms, most commonly non-nociceptive somatic sensations such as paresthesias, vibrations, or feelings of heaviness, as well as auditory disturbances (e.g., hypoacusis) and visual illusions (Ostrowsky et al., 2002). Gustatory alterations have been reported in a single case described in the literature (Mulak et al., 2008). In our cohort, sensory symptoms occurred more frequently than emotional or visceral semiologies; however, when present, they were rarely recognized by patients as components of their habitual seizures. Temporopolar DCS in our study evoked a broad range of sensory experiences, including non-nociceptive somatic sensations, vestibular sensations, auditory disturbances, and visual illusions. These findings further support a network-level mechanism, whereby stimulation of the temporal pole produces sensory phenomena through propagation to primary sensory cortices, posterior temporal and parieto-insular regions, or visual pathways.

A considerable proportion of our study cohort reported brief, painful, electric shock–like sensations in the face or teeth ipsilateral to the side of DCS. These sensations were strictly time-locked to stimulation and were in no case a part of the patients' habitual seizures. We hypothesize that this effect may result from activation of ipsilateral trigeminal sensory pathways due to current spread via volume conduction near the skull base and adjacent trigeminal fibers. Pain is generally not evoked by DCS unless applied to the primary sensory cortex or posterior insula (Montavont et al., 2015), or via electrode contacts in close proximity to the dura mater (Fontaine et al., 2018). An intense pain sensation during temporopolar DCS has been described in a single case report (Swanson et al., 2021): Electrical stimulation of the left temporal pole in a patient with a temporopolar encephalocele induced burning, lancinating pain in the left cheek for the duration of the stimulus train, accompanied by subtle, rapid twitching of the left lower eyelid. The authors proposed that the pain arose from direct stimulation of the trigeminal nerve located just inferior to the encephalocele, while the eyelid twitching likely resulted from activation of the left m. orbicularis oculi via connections between distal branches of the trigeminal and facial nerves. A similar mechanism, likely involving activation of the facial nerve, may underlie DCS-induced twitching of the ipsilateral mouth corner, which was observed in five patients in our cohort.

Cognitive symptoms during temporopolar DCS have been reported only rarely, with involuntary memory recall described in isolated cases (Curot et al., 2017). In contrast, language disturbances have been more commonly observed. Mariani et al. (2021) reported language impairments in eight patients following stimulation of the dominant hemisphere, most often involving DCS in the lateral temporal pole (7/8 cases). Additionally, a single case report described impaired naming of faces, living entities, and body parts during stimulation of the basal temporal pole (Manjarrez-Garduño et al., 2018), consistent with evidence implicating the temporal pole in language processing and naming functions (Tranel, 2009; Abel et al., 2015).

In our cohort, we identified seven cases of cognitive disturbances, including memory flashbacks, déjà vu experiences, and subjective difficulties thinking, which were predominantly recognized by patients as their typical ictal manifestations. Notably, all of these symptoms were elicited by DCS of the mesial temporal pole. In contrast, no language impairments were observed in our cohort; this finding likely reflects the limited stimulation of the lateral temporal pole, as language disturbances have been shown to be predominantly associated with stimulation of this region.

From a clinical perspective, our findings have important implications for the interpretation of DCS-induced symptoms during invasive presurgical evaluation. The substantial overlap between temporopolar DCS semiology and that of other temporal lobe structures limits the localizing value of stimulation-induced symptoms arising from the temporal pole. In particular, visceral and emotional phenomena should be interpreted with caution and should not be considered reliable indicators of temporopolar seizure onset when guiding surgical decision-making. For example, although a temporal pole lesion such as an encephalocele may represent the primary site of seizure onset (Morone et al., 2015; Agashe et al., 2023), the presence of a differently located epileptogenic focus cannot be excluded. Recent evidence has increased the clinical utility of SEEG in cases of encephaloceles, as it may help avoid unnecessary resections, enable sparing of mesial temporal structures, and reduce the risk of postoperative neuropsychological decline (Panov et al., 2016; de Souza et al., 2018; Dahal et al., 2023).

Several limitations should be acknowledged. First, although the cohort size was appropriate, stimulation parameters were not fully uniform across patients and were determined by clinical considerations. Second, interindividual variability in electrode placement constitutes an additional limitation. Finally, current spread during electrical stimulation, together with the close anatomical proximity of temporopolar contacts to mesial temporal structures, may confound the attribution of certain elicited symptoms specifically to the temporal pole.

In conclusion, DCS of the human temporal pole elicits a broad yet non-specific spectrum of clinical symptoms that substantially overlaps with those evoked by stimulation of neighboring temporal lobe structures. These findings support the view of the temporal pole as an integrative hub within temporolimbic networks, rather than a region characterized by distinct stimulation-induced phenomena. Future studies should adopt network-oriented approaches that combine DCS with functional connectivity analyses and high-resolution tractography to further elucidate the role of the temporal pole within epileptogenic and cognitive networks.

## CRediT authorship contribution statement

**Yulia Novitskaya:** Data curation, Formal analysis, Methodology, Visualization, Writing – original draft. **Andreas Schulze-Bonhage:** Conceptualization, Writing – review & editing.

## Declaration of competing interest

Y.N. received support from Eisai and Angelini Pharma outside of the present work. A.S-B. received research support from Precisis and UNEEG, and personal honoraria for lectures or advice from Angelini Pharma, BIAL, Eisai, Jazz Pharma, Precisis, UCB, and UNEEG, all outside of the present work.
